# Swept-source Optical Coherence Tomography Angiography in a Patient with Bietti Crystalline Dystrophy Followed for Ten Years

**DOI:** 10.4274/tjo.galenos.2018.90768

**Published:** 2019-04-30

**Authors:** Şefik Can İpek, Ziya Ayhan, Sibel Kadayıfçılar, Ali Osman Saatci

**Affiliations:** 1 Dokuz Eylül University Faculty of Medicine, Department of Ophthalmology, İzmir, Turkey; 2 Hacettepe University Faculty of Medicine, Department of Ophthalmology, Ankara, Turkey

**Keywords:** Bietti crystalline dystrophy, optical coherence tomography, optical coherence tomography angiography, retinal dystrophy

## Abstract

A woman with Bietti’s crystalline dystrophy (BCD) was first examined when she was 27 years of age and has been followed for 10 more years. The disease course was monitored initially with spectral domain-optical coherence tomography and then with swept-source optical coherence tomography angiography (OCTA). OCTA analysis showed that choroidal vessels could be visualized at the outer retinal layer segmentation due to retinal pigment epithelial atrophy and blood flow was reduced at the level of choroidal segmentation. OCTA can play a major role in the follow-up of BCD patients by analyzing changes in choroidal flow.

## Introduction

Bietti crystalline dystrophy (BCD) is a retinal dystrophy characterized by shiny yellow crystalline deposits in the retina and sometimes the limbus with progressive chorioretinal atrophy starting in the posterior pole.^1^ Mutations in the *CYP4V2* gene have been detected in patients with BCD.^2^

Indocyanine green angiography, one of the conventional imaging methods, shows extensive areas of hypofluorescence due to choriocapillaris atrophy as the disease progresses in posterior pole.^3^ Optical coherence tomography has demonstrated reduced central foveal and subfoveal thickness, hyperreflective spots in all retinal layers and even within the choroid, outer retinal tubulations, and hyperreflective plaques in the retinal pigment epithelium (RPE)-Bruch’s membrane complex.^4^

Compared to conventional methods, there are fewer reports of findings associated with BCD in optical coherence tomography angiography (OCTA), which is a very new imaging modality. In this case report, we describe the clinical entities and OCTA characteristics of a woman with BCD who was followed for 10 years.

## Case Report

**Figure 1:**
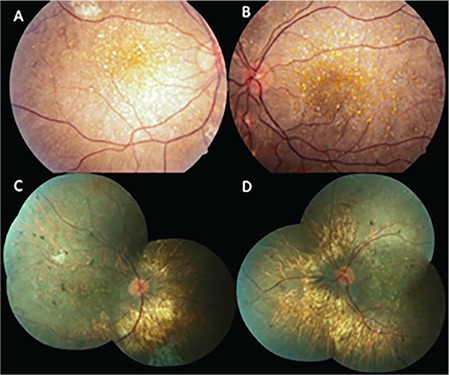
Color fundus images: In 2008, extensive intraretinal crystals concentrated around the posterior pole and relatively little chorioretinal atrophy were observed in the right (A) and left (B) eyes; In 2018, there was a marked decrease the number of crystalline deposits and increase in chorioretinal atrophy in the right (C) and left (D) eyes

A 27-year-old woman presented to our clinic in 2008 with progressive visual impairment in both eyes. On ophthalmologic examination, her best corrected visual acuity (BCVA) on Snellen chart was 0.3 (-4.50) in the right eye and 0.2 (-4.50) in the left eye. Slit-lamp examination showed clear cornea, calm anterior chamber, and transparent lens in both eyes. Deposits were not observed in the corneal limbus of either eye. The optic discs appeared normal on fundus examination. Extensive shiny white-yellow deposits were observed in the posterior pole and mid-peripheral retina (Figures 1A and B). Based on these findings, a clinical diagnosis of BCD was made and the patient was scheduled for follow-up.

**Figure 2:**
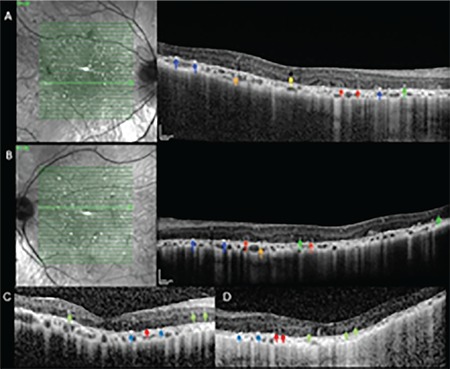
Enhanced depth imaging spectral domain-optical coherence tomography images from 2014, right (A) and left (B) eye. Red arrows: Outer retinal tubulations; Blue arrows: Hyperreflective plaque-like deposits in the retinal pigment epithelium (RPE)-Bruch’s membrane; Green arrows: Intraretinal hyperreflective spots; Orange arrows: Choroidal hyperreflective spots; Yellow arrow: Intraretinal cystic space. Swept-source optical coherence tomography images from 2018, right (C) and left (D) eye. Hyperreflective plaque-like accumulations on the RPE-Bruch’s membrane (blue arrow), outer retinal tubulations (red arrow), and hyperreflective intraretinal spots (green arrow)

Upon retrospective analysis of her records, we noticed that patient did not undergo OCT in 2008. OCT (Spectralis; Heidelberg Engineering, Heidelberg, Germany) performed in 2014 revealed central macular thickness was 194 µm in the right eye and 198 µm in the left eye. Hyperreflective intraretinal spots were observed in the sensorial retina and hyperreflective plaque-like deposits were identified at the RPE-Bruch’s membrane junction. Intraretinal cystic spaces were observed in some of the sections. Outer retinal tubulation was noted in the outer retinal layers. Choriocapillaris atrophy and subsequent enhanced visibility of the large choroidal vessels was noted in enhanced depth imaging mode, which provides better visualization of the choroid. Complete obliteration of the choroidal vasculature was observed in some places. Choroidal hyperreflective foci were noted around the choroidal vessels (Figures 2A and B). Swept-source OCT (SS-OCT) performed in 2018 showed a relative reduction in the intraretinal hyperreflective spots and hyperreflective plaque-like deposits at the RPE–Bruch’s membrane detected in 2014 (Figure 2C and D).

**Figure 3:**
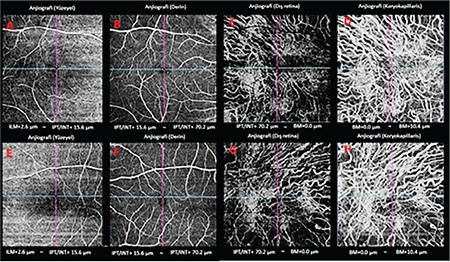
Spectral domain-optical coherence tomography angiography images, 2018: from left to right, projections of the superficial, deep, outer retinal, and choriocapillaris in the right (A-D) and left (E-H) eye. The choroidal vasculature is evident in the area of the outer retina layer projection due to increased permeability secondary to retinal pigment epithelium atrophy, but no flow associated with the choriocapillaris can be seen in the region corresponding to the choriocapillaris projection

On examination in 2018, BCVA was 0.1 (-4.50) in both eyes. There were still no signs of corneal pathology on slit-lamp examination. Fundus examination revealed retinal crystalline deposits and extensive areas of retinal and choroidal atrophy in both eyes (Figure 1C and D). In SS-OCTA (Topcon DRI OCT Triton, Topcon, Japan), the choroidal vessels were visible in the area of outer retinal layer projection due to increased permeability resulting from diffuse RPE atrophy; in the area corresponding to the choriocapillaris projection, no flow associated with the choriocapillaris was observed and vessels belonging to the deeper choroidal layers were apparent in this area (Figure 3A, B, C, and D [right eye]; E, F, G, and H [left eye]).

## Discussion

Hirashima et al.^5^ used SS-OCTA to evaluate 9 eyes of 9 patients with BCD, 16 eyes of 16 retinitis pigmentosa patients with *EYS* mutation, and 16 eyes of 16 control subjects. The outer choroidal vascular area was 43.34±5.76% in eyes with BCD, 53.73±4.92% in eyes with *EYS* mutation/retinitis pigmentosa, and 52.80±4.10% in healthy subjects, with the value in BCD being significantly lower. Thinning in the outer choroidal vascular area in eyes with BCD was associated with thinning of the subfoveal choroid, and interestingly, the inner choroidal vessels could not be identified in 8 of the 9 BCD eyes.

Miyata et al.^6^ analyzed 13 eyes of 13 patients with BCD using Optovue OCTA (RTVue XR, Avanti-AngioVue, Optovue, Freemont, CA, USA) and demonstrated reduced choriocapillaris flow in 12 (92%) of the 13 eyes. The authors reported that subfoveal choriocapillaris thickness was correlated with visual acuity.

In our patient, after 10 years of follow-up, we observed a slight decline in vision, which was already poor at baseline, reduction of intraretinal crystals, and substantial progression of chorioretinal atrophy. Consistent with the publications cited above, we noted a significant decrease in choriocapillaris flow in SS-OCTA performed 10 years after the first examination.

We believe that OCTA will become an important adjunctive examination in the follow-up of choroidal blood flow and changes in the choroidal vasculature in BCD, a disease that causes progressive vision loss.
